# Nanotechnology-Based Strategy for Enhancing Therapeutic Efficacy in Pancreatic Cancer: Receptor-Targeted Drug Delivery by Somatostatin Analog

**DOI:** 10.3390/ijms25105545

**Published:** 2024-05-19

**Authors:** Xin Gu, Joydeb Majumder, Olena Taratula, Andriy Kuzmov, Olga Garbuzenko, Natalia Pogrebnyak, Tamara Minko

**Affiliations:** 1 Department of Pharmaceutics, Ernest Mario School of Pharmacy, Rutgers, The State University of New Jersey, Piscataway, NJ 08554, USA; 2 Department of Pharmaceutical Sciences, College of Pharmacy, Oregon State University, Portland, OR 97201, USA; 3 Rutgers Cancer Institute of New Jersey, Rutgers, The State University of New Jersey, New Brunswick, NJ 08901, USA

**Keywords:** somatostatin receptor 2 (SSTR2), nanoparticle-based drugs, liposome, paclitaxel, targeted delivery system

## Abstract

A novel nanotechnology-based drug delivery system (DDS) targeted at pancreatic cancer cells was developed, characterized, and tested. The system consisted of liposomes as carriers, an anticancer drug (paclitaxel) as a chemotherapeutic agent, and a modified synthetic somatostatin analog, 5-pentacarbonyl-octreotide, a ligand for somatostatin receptor 2 (SSTR2), as a targeting moiety for pancreatic cancer. The cellular internalization, cytotoxicity, and antitumor activity of the DDS were tested in vitro using human pancreatic ductal adenocarcinoma (PDAC) cells with different expressions of the targeted SSTR2 receptors, and in vivo on immunodeficient mice bearing human PDAC xenografts. The targeted drug delivery system containing paclitaxel exhibited significantly enhanced cytotoxicity compared to non-targeted DDS, and this efficacy was directly related to the levels of SSTR2 expression. It was found that octreotide-targeted DDS proved exceptionally effective in suppressing the growth of PDAC tumors. This study underscores the potential of octreotide-targeted liposomal delivery systems to enhance the therapeutic outcomes for PDAC compared with non-targeted liposomal DDS and Paclitaxel-Cremophor^®^ EL, suggesting a promising avenue for future cancer therapy innovations.

## 1. Introduction

Accounting for 90% of all pancreatic malignancies, pancreatic ductal adenocarcinoma (PDAC) is the fourth leading cause of cancer-related death in the United States, with a five-year survival rate of 8% [1]. Although surgery can be an effective way to treat PDAC, the statistical report suggests that 80% of pancreatic cancer cases are unresectable, and, in many cases, local recurrence can occur after tumor resection [2]. PDAC’s complexity is underscored by its genetic diversity, elevated interstitial fluid pressure, and thick tumor stroma, which contribute to a challenging microenvironment. These factors notably amplify its tendency to metastasize and resist drugs, making it difficult for conventional chemotherapy treatments to deliver therapeutic agents effectively [3,4,5]. Nanotechnology has risen as a critical field in tackling the complexities of diverse cancer types through innovative therapeutic and diagnostic approaches [6,7,8,9]. The versatility and potential of this domain have led to its growing prominence, especially in improving treatments for conditions such as PDAC, making substantial progress in recent years [10]. The FDA’s approval of nanoformulations like Onivyde^®^ (irinotecan liposome) and Abraxane^®^ (nab-paclitaxel) for PDAC treatment emphasizes the impactful role of nanotechnology. These advanced treatments have outperformed conventional gemcitabine-based therapies in clinical effectiveness [11,12]. However, it is essential to note that these formulations do not specifically target unique cell markers, which suggests an area for further innovation and development in nanoparticle-based therapies.

**Figure 1:**
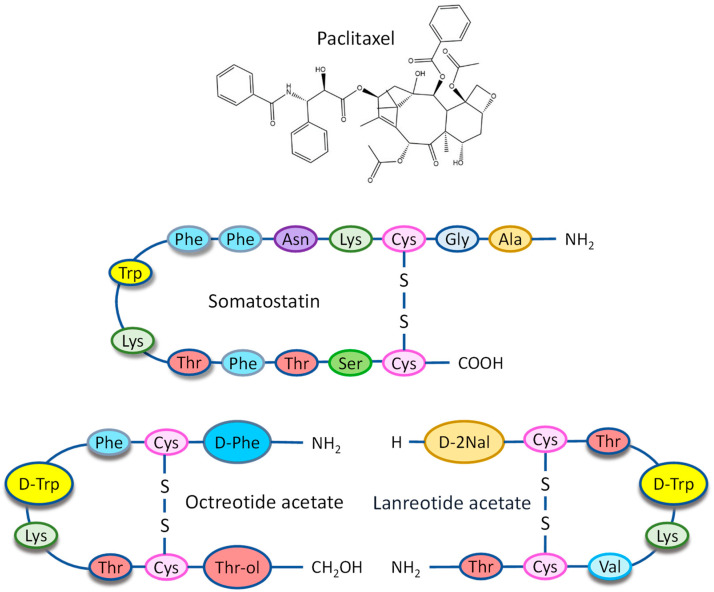
Paclitaxel’s structural formula and the amino acid compositions of human somatostatin-14 and its synthetic analogs octreotide and lanreotide. Somatostatin (SST) is presented in two forms of cyclic peptides, 14 and 28 amino acids. The somatostatin synthetic analogs, octreotide, and lanreotide, have 8 amino acids. The four amino acids (Phe, (D)Trp, Lys, Thr) necessary for binding to the SSTR2 receptor are shadowed. The terminal Thr-COOH group in SST analogs is reduced to an alcoholic group, while L-Tryptophan has been replaced by the non-natural enantiomer D-Tryptophan, in order to limit enzymatic degradation and increase the peptide’s biological activity.

Liposomes, a type of phospholipid-based vesicles that offer the ability to encapsulate hydrophilic drugs in their internal core and lipophilic drugs within their lipid bilayers, leading to the enhanced solubility and encapsulation efficiency of lipophilic medications [13]. Since their discovery, they have proven to be a productive delivery system for hydrophobic drugs with superior biological compatibility. Moreover, liposomes can evade the reticuloendothelial system (RES) through surface modifications, such as PEG conjugation [14,15,16]. Additionally, active targeting can be achieved by coupling ligands to cell surface receptors. With their precision engineering, nanoparticles can target tumor-supporting cancer-associated fibroblasts (CAFs) and distinct markers on PDAC cells, such as epidermal growth factor receptor (EGFR), vascular endothelial growth factor receptor (VEGFR), integrins, hyaluronan, transferrin, etc. This targeted approach significantly improves the specificity and effectiveness of treatments [10,17,18,19,20]. This targeted approach improves therapeutic outcomes and minimizes damage to healthy cells, reducing the severe side effects typically caused by conventional chemotherapy and radiation. Apart from the markers mentioned above, it has been found that somatostatin receptor 2 (SSTR2) is abundantly expressed in PDAC cells [21,22,23,24,25,26,27]. Hence, it may be an attractive target for the related ligand—somatostatin—and its analogs, such as octreotide and lanreotide (Figure 1). Derived primarily from the bark of the *Taxus brevifolia* tree, Paclitaxel (Figure 1) is a potent anti-cancer medication that the FDA has approved for the treatment of ovarian, breast, and non-small cell lung cancer (NSCLC), as well as Kaposi’s sarcoma. Its effectiveness in treating these types of cancer is well established. Moreover, it has also been used off-label to address solid tumors, including advanced cervical cancer and metastatic bladder cancer [28,29]. However, commercially available paclitaxel is mainly in the form of Taxol^®^ (Paclitaxel-Cremophor^®^ EL) and Abraxane^®^ (nab-paclitaxel), which cannot be explicitly delivered to the tumor site; therefore, they restrict treatment efficacy and increase the risk of damage to healthy organs. In this study, a modified synthetic somatostatin (SST) analog, 5-pentacarbonyl-octreotide (OCT), was used as an SSTR2-targeting agent, and agent-conjugated liposomal paclitaxel, OCT-DSPE-PEG-Liposome -Paclitaxel (OCT-Lip-PTX), is proposed as a potential model drug for the formulated targeting therapy.

## 2. Results

### 2.1. Synthesis and Characterization of Liposomal Formulations

**Figure 2:**
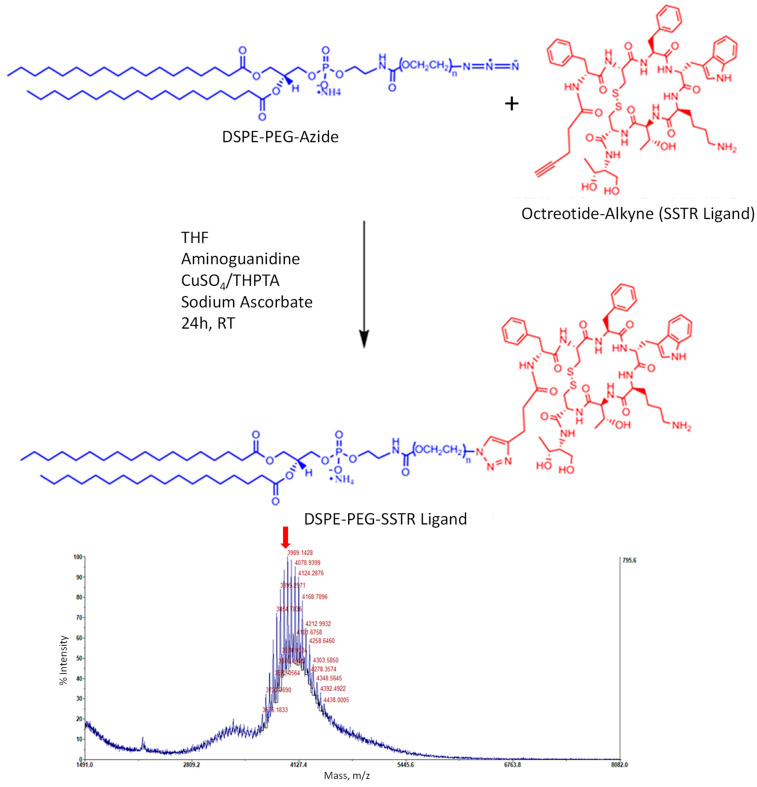
Schematic representation of DSPE-PEG-SSTR ligand synthesis (**top panel**) and representative image of the final product’s MALDI-TOF-MS (**bottom panel**). A copper-catalyzed azole-alkyne click chemistry (CuAAC) reaction was performed to conjugate DSPE-PEG-azide with 5-pentynecarbonyl-octreotide (OCT). Both substances were dissolved in tetrahydrofuran (THF) and mixed. After that, the mixture of CuSO4 and tris (benzyltriazolylme-thyl) amine (THPTA) was added dropwise to the reaction mixture and stirred. Next, aminoguanidine and sodium ascorbate were added to the reaction mixture and stirred for 24 h at room temperature (RT). The final product was lyophilized to get the DSPE-PEG2000-OCT ligand in solid powder form. Matrix-assisted laser desorption/ionization time-of-flight mass spectrometry (MALDI-TOF-MS) confirmed the final yield of the ligand. The predicted mass of the resulting compound calculated based on the information provided by manufacturers (with accounting on the polydispersity of PEG), is shown at the MALDI-TOF-MS image as red arrow.

**Figure 3:**
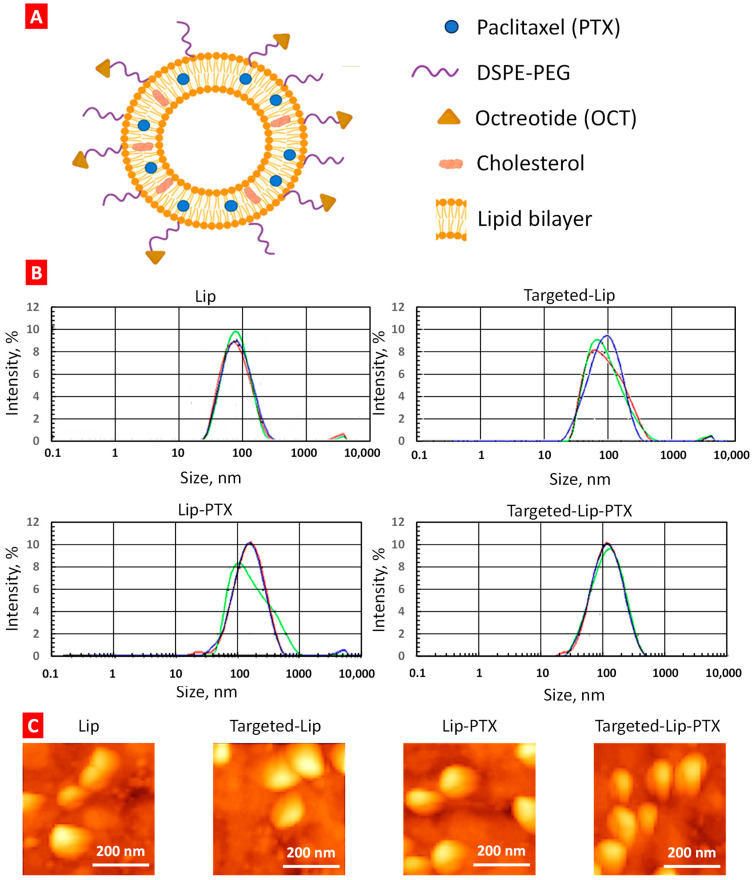
The structure, size distribution and atomic force microscopy (AFM) images of the formulation. (**A**) The assumed structure of the targeted liposomal delivery system containing paclitaxel. (**B**) The size distribution of empty liposomes (Lip), targeted liposomes without drugs (Targeted-Lip), non-targeted liposomes containing paclitaxel (Lip-PTX), and targeted liposomes containing paclitaxel (Targeted-Lip-PTX). The particle size of liposomes was measured by dynamic light scattering using Malvern Zetasizer Nano (Malvern Instruments, UK) Different colors show distribution curves for three individual measurements of the same formulation. (**C**) Representative AFM images of liposomal formulations. Panoramic images were captured in phase contrast mode.

**Figure 4:**
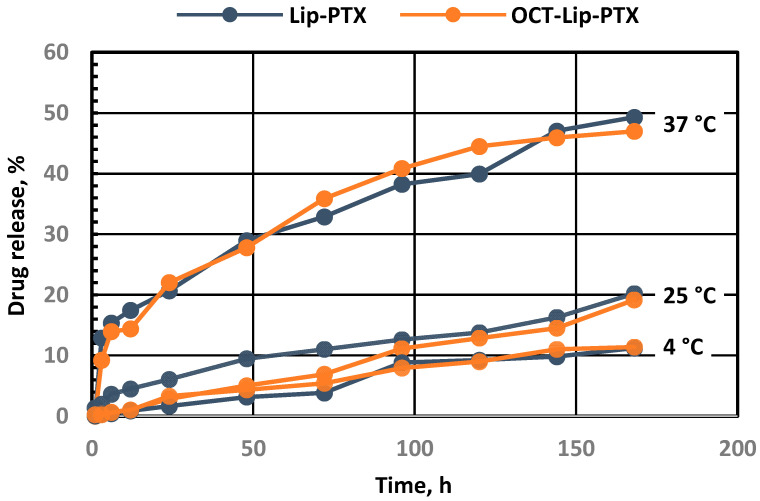
In vitro drug release profiles of liposomal formulations. Non-targeted (Lip-PTX) and cancer-targeted (OCT-Lip-PTX) nanoparticles were introduced into release media that were maintained at three constant temperatures, 4 °C, 25 °C, and 37 °C, over 7 days. Samples were collected at specific time points of 0 h, 0.5 h, 1 h, 2 h, 3 h, 6 h, 8 h, 12 h, 24 h, 48 h, 72 h, 96 h, 120 h, 144 h, and 168 h. After sample collection, an equal volume of 1% Tween 20/PBS solution was replaced at each time point. Free paclitaxel released from the formulations was separated by dialysis, and the bound drug concentration in the liposomes was analyzed using HPLC methods.

The size, type and composition of liposomes were selected based on our previous investigations in order to provide the most effective delivery to cancer cells and of the maximal anticancer drug efficacy in vivo [6,9,30,31]. DSPE-PEG_2000_-OCT was synthesized using the methods described below; matrix-assisted laser desorption/ionization time-of-flight mass spectrometry (MALDI-TOF-MS) confirmed the final yield. Data were acquired by ABI-MDS SCIEX 4800 system (SCIEX, Framingham, MA, USA) with linear mid-mass mode from 1.5 kDa to 8 kDa (Figure 2). Figure 3 illustrates the structure of OCT-Lip-PTX, with paclitaxel entrapped between the hydrophobic tails and octreotide linked to liposomes through DSPE-PEG_2000_. Data from the dynamic light scattering (DLS) procedure show that all liposome formulations had sizes of 80 nm to 140 nm (Figure 3), with a slight negative charge (Table 1). It was found that OCT-Lip-PTX showed a comparable releasing profile at various temperatures to its non-targeting counterpart (Figure 4).

### 2.2. Expression of SSTR2 in Pancreatic Cancer Cells

**Figure 5:**
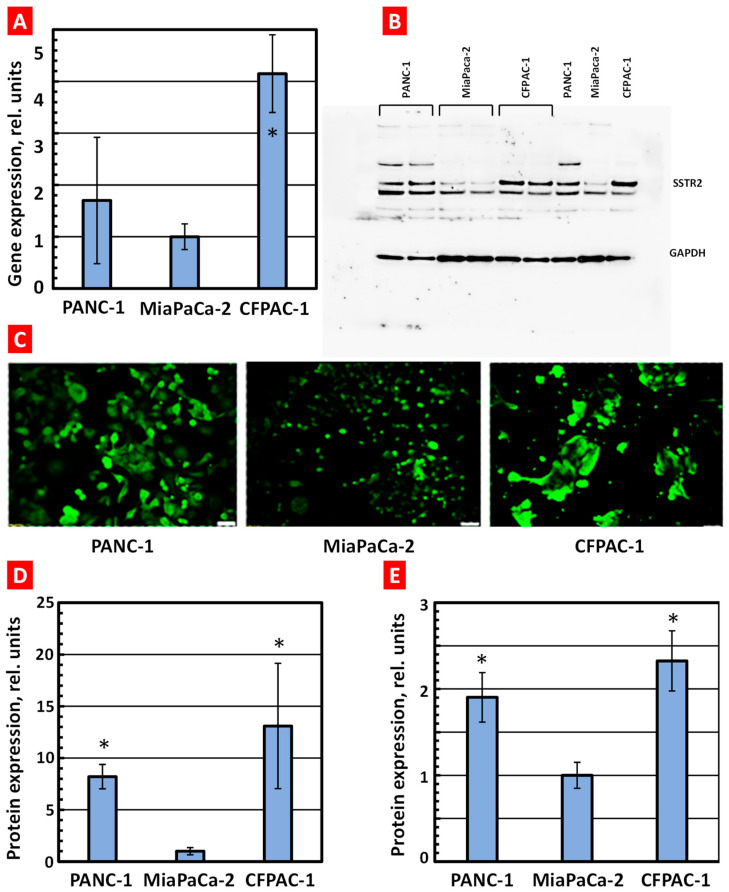
Expression of somatostatin receptors 2 (SSTR2) in PANC-1, MiaPaca-2, and CFPAC-1 human pancreatic cancer cells. (**A**) Gene expression measured by QRT-PCR. (**B**) Representative image of protein expression measured by Western blotting. GAPDH was used as an internal standard. (**C**) Representative image of protein expression measured by immunohistochemistry (scale bars—100 µm). Pancreatic cancer cells were incubated for 24 h with Anti-SSR2antibody (ab9550) followed by Goat Anti-Rabbit IgG H&L (Alexa Fluor^®^ 488, ab150077, green fluorescence). (**D**) Quantitative analysis of Western blotting image. (**E**) Quantitative analysis of immunohistochemistry images. The expression of SSTR2 mRNA/protein in MiaPaca-2 cells was set to 1 unit. Means ± SD are shown. * *p* < 0.05 when compared with MiaPaca-2 cells.

The results of quantitative reverse transcription polymerase chain reaction (QRT-PCR) assays demonstrated that SSTR2 mRNA is expressed in all three types of investigated pancreatic cancer cell lines, with varying degrees of abundance. It is noteworthy that the expression of SSTR2 was found to be considerably different among the three cells. Specifically, CFPAC-1 cells exhibited almost 4.2-fold higher mRNA expression than MiaPaca-2 cells and 2.5-fold higher mRNA expression than PANC-1 cells (Figure 5). All RNA samples tested positive for internal controls GAPDH and β-Actin.

Immunocytochemistry assays were performed to confirm the SSTR2 protein expression qualitatively. All three types of paraformaldehyde-fixed cells were immunostained, and the expression of SSTR2 was observed in CFPAC-1, MiaPaca-2, and PANC-1. CFPAC-1 showed the strongest fluorescence expression among the three cell lines (Figure 5). Then, SSTR2 protein expression was assessed quantitatively by Western blot in cell lysate samples. A significant amount of the expression of endogenous SSTR2 was observed in CFPAC-1 cells in the range of 75 kDa, and a moderate amount of SSTR2 protein was observed in PANC-1 cells compared to MiaPaca-2 cells, which was at a barely visible level (Figure 5). The immunocytochemistry and Western blot results were consistent with the RT-qPCR results, indicating that SSTR2 is much more pronounced in CFPAC-2 cells.

### 2.3. Cellular Internalization of Liposomes and Intracellular Release of Paclitaxel

**Figure 6:**
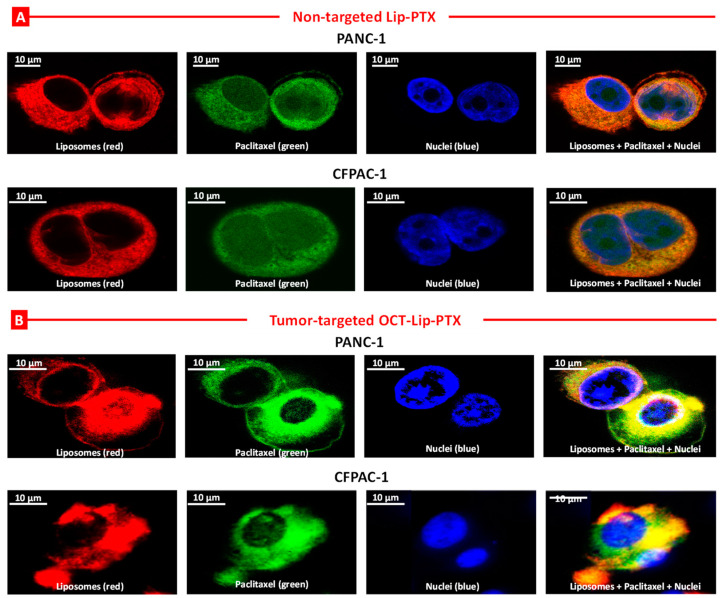
Cellular internalization of non-targeted (Lip-PTX (**A**)) and tumor-targeted (OCT-Lip-PTX (**B**)) liposomes containing paclitaxel by a confocal microscope in Human PANC-1 and CFPAC-1 pancreatic cancer cells. The cells were incubated for 24 h with liposomes (red fluorescence) containing paclitaxel (green fluorescence). Nuclei were labeled with DAPI (blue fluorescence). The superimposition of red and green colors gives yellow or orange colors.

The results of the confocal microscope clearly indicated that fluorescence liposomes (red fluorescence) were internalized into the pancreatic cancer cells and localized in the cytoplasm; paclitaxel (green fluorescence) was successfully released from liposomes and localized in the cytoplasm and nuclei (blue fluorescence). Both non-targeted (Lip-PTX) and cancer-targeted (OCT-Lip-PTX) liposomal formulations were internalized by the pancreatic cancer cells and released their PTX payload in the cytoplasm (Figure 6). The fluorescence of labeled PTX tightly packed in liposomes was quenched [32]. Therefore, the appearance of the green fluorescence signal inside the cells testified the release of the drug from liposomes inside the cells. The accumulation of paclitaxel in the cytoplasm facilitated its pharmacological activities, such as microtube targeting.

### 2.4. Cytotoxicity of Different Formulations of Paclitaxel

**Figure 7:**
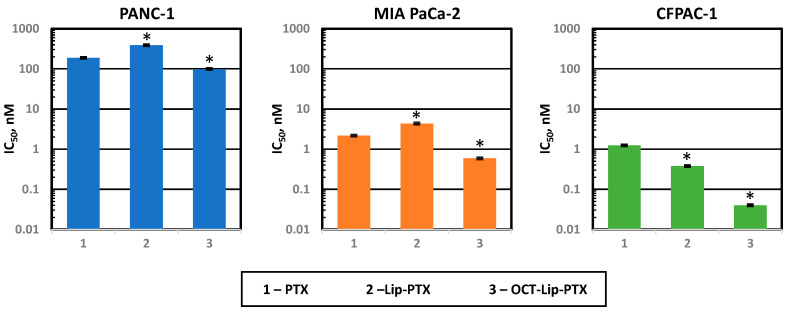
Cytotoxicity of free paclitaxel (PTX), non-targeted (Lip-PTX) and targeted by octreotide (OCT-Lip-PTX) liposomal PTX formulations in different human pancreatic cancer cells. Means ± SD are shown. * *p* < 0.05 when compared with free unbound paclitaxel.

The IC_50_ values of free non-bound paclitaxel, PEGylated liposomal paclitaxel, and octreotide-targeted PEGylated liposomal paclitaxel against PANC-1, MiaPaca-2, and CFPAC-1 cells showed the different cytotoxicity of different treatments for all cell lines (Figure 7). After 72 h incubation, the octreotide-conjugated liposomal paclitaxel concentration for 50% inhibition (IC_50_) of CFPAC-1 cells was approximately 0.04 nM, which was significantly lower than that of free paclitaxel and PEGylated liposomal paclitaxel, with the mean IC_50_ equaling 1.24 nM and 0.38 nM, respectively. Similar results were obtained for both MiaPaca-2 and PANC-1. The average IC_50_ of octreotide-conjugated PEGylated liposomal paclitaxel and paclitaxel against PANC-1 was 98.95 nM and 188.40 nM, respectively. For MiaPaca-2, the IC_50_ for octreotide-conjugated PEGylated liposomal paclitaxel and paclitaxel was 0.59 nM and 2.17 nM, respectively.

### 2.5. Comparison of the Antitumor Effect of Free Paclitaxel, Non-Targeted and Pancreatic Cells Targeted Liposomal Formulations of Paclitaxel

**Figure 8:**
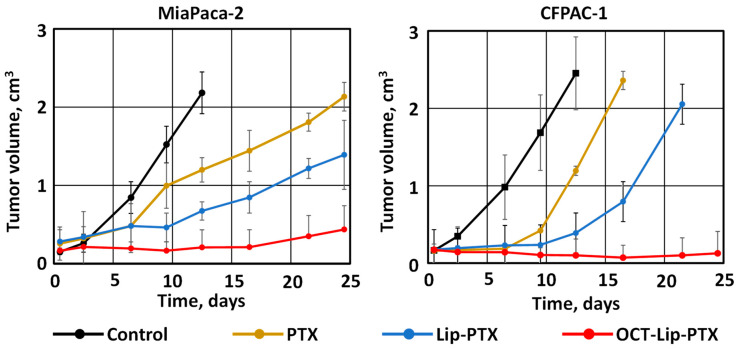
Antitumor activity in mice bearing xenografts of MiaPaca-2 and CFPAC-1 human pancreatic cancer cells. After tumors reached a size of about 0.2 cm^3^ (approximately eight days after the subcutaneous injection of cancer cells), mice were treated twice per week within four weeks with the following formulations: Control (mice treated with saline), PTX (Paclitaxel-Cremophor^®^ EL), Lip-PTX (DSPE-PEG-Liposome-Paclitaxel), and OCT-Lip-PTX (5-pentacarbonyl-octreotide)-DSPE-PEG-Liposome-Paclitaxel). Mice were euthanized when the tumor size exceeded 2 cm^3^ or at the end of the treatment. Means ± SD are shown.

The successful uptake of liposomal paclitaxel by PDAC cells and promising in vitro results do not necessarily guarantee exceptional tumor growth suppression in vivo. Therefore, the next step of the present study was focused on investigating the advantages of DDS targeted to somatostatin receptors 2 overexpressed in pancreatic cancer cells when compared with a non-targeted delivery system and clinically used Taxol^®^ (Figure 8). The mice inoculated with MiaPaca-2 and CFPAC-1 cells were treated with the commercially available paclitaxel (PTX), non-targeted liposomal paclitaxel—Lip-PTX—and targeted to SSTR2 OCT-Lip-PTX. All the investigated drug formulations were used in the previously detected maximum-tolerated dose of paclitaxel (MTD, 2.5 mg/kg). The data obtained in the series of mice inoculated with cancer cells demonstrated that the administration of free paclitaxel and untargeted Lip-PTX limited tumor growth. In contrast, the mice with the same type of tumor that were treated with a targeted drug delivery system (OCT-Lip-PTX) demonstrated significantly better outcomes, resulting in the prevention or almost complete elimination of tumors. Similar results were obtained for both MiaPaca-2 and CFPAC-1 types of human pancreatic tumor. OCT-Lip-PTX demonstrated superior efficacy in eliminating PDAC cells, suppressing pancreatic tumor growth, and even reducing tumor volume, compared to the non-targeted liposome formulations currently used in clinic Taxol^®^. It also should be stressed that CFPAC-1 cells were more resistant to paclitaxel than CFPAC-1 and MiaPaca-2 cells. However, the positive effects of paclitaxel delivery by non-targeted and especially OCT-targeted liposomes were more pronounced in these cells.

## 3. Discussion

The ligand–target SSTR2, chosen for this study, has the potential to enhance potency and minimize undesirable side effects. The studies have shown that the SSTR2 is highly expressed in PDAC cells and tissues, although there may be variations in findings across different studies [21,22,23,24,25,26,27,33,34,35]. This variability could be due to differences in measurement techniques, experimental conditions, or treatment methods with corresponding ligands. SSTRs have been identified in a range of neuroendocrine tumors, such as pituitary adenomas, pancreatic endocrine tumors, small cell lung cancers, medullary thyroid carcinomas, paragangliomas, and carcinoids. Most studies concluded that the level of SSTR2 expression in the tissues affected by pancreatic cancer is considerably higher than that of the adjacent healthy tissues. In particular, it was found that more than 80% of studied patients (88 out of 108) were SSTR2-positive [23]. This expression in SSTR2-positive cancer tissues was 8 to 10 times higher than in non-cancerous pancreatic tissue. Although the clinical relevance of such overexpression is unclear, the difference in the expression in cancerous and normal tissue creates the preconditions for the use of SSTR2 ligands as an effective targeting moiety to provide a cancer-specific delivery of therapeutics and the possible limitation of adverse side effects upon healthy tissues.

Notably, tumors that exhibit neuroendocrine traits and significant SSTR2 expression could be potential candidates for peptide receptor radionuclide therapy (PRRT), either as a standalone treatment or in combination with other therapies like targeted chemotherapy [36,37,38]. This finding presents exciting treatment possibilities for a specific subset of pancreatic tumors, potentially transforming the management of this condition. Octreotide, a frequently used octapeptide analog of SST, demonstrates a higher binding affinity towards SSTR2 and SSTR5 and an extended half-life of 2 h, in contrast to the short half-life of the native SST, which ranges from 1–3 min. SST activates SHP-1, triggers intracellular pro-apoptotic signals, and promotes apoptosis [39]. This makes octreotide—the first somatostatin analog approved for treating hormone-producing pituitary, pancreatic, and intestinal neuroendocrine tumors—a potential candidate for the tumor-targeted delivery of drugs, highlighting the practical implications of the research [40].

However, previous clinical evaluations demonstrated that the impact of SST analogs on symptom management and decelerating tumor growth in neuroendocrine tumors (NETs) is still limited [41,42]. Despite this, SST analogs have shown substantial potential to be combined with other therapeutic or imaging agents via bioconjugation for the targeted delivery of its payloads to SSTR-positive cancer cells. Sun et al. proposed a targeted delivery system by conjugated cytotoxic camptothecin (CPT) with an SST analog (JF-07-69) via an activated carbamate linker, which demonstrated a 92% inhibition rate in CFPAC-1 tumor models after 4-week treatment and a 56% inhibition rate at low treatment doses (1 mg/pellet) [25]. Further studies involved PTX-OCT conjugates with additional modifications, such as PTX-Lys-OCT, PTX-Phe-OCT, and PTX-Phe-OCT-Lys-PTX, incorporating a succinyl linker [42]. The paclitaxel–octreotide demonstrated significant tumor growth inhibition and reduced microvessel density in A549 NSCLC xenograft models, indicating potent anti-angiogenic effects alongside direct antitumor activity. Conjugating PTX with OCT via a hydrophilic PEG linker significantly enhanced the solubility and SSTR-binding affinity of the conjugate, thereby increasing cytotoxicity against SSTR-overexpressing NCI-H446 cells and demonstrating superior in vivo efficacy and reduced systemic toxicity in xenograft models compared to commercial PTX formulations [43]. However, the targeted delivery of paclitaxel via nanoparticle integration remains limited, but recent research has demonstrated superior antitumor effects in tumor xenograft models. This study proposed a targeting liposome drug delivery system by connecting octreotide to paclitaxel-loaded liposomes via DSPE-PEG linkers for treating SSTR2-positive PDAC tumors.

Our research centered on a novel drug delivery system designed to target tumor cells, explicitly using the abovementioned targeting system. This system employs a dual-targeting strategy that combines the enhanced permeability and retention (EPR) effect for passive targeting with ligand–receptor interactions for active targeting, specifically using octreotide to target SSTR2. The delivery vehicle we developed is an octreotide-DSPE-PEG-liposomal formulation loaded with paclitaxel, engineered to maximize drug delivery efficiency to tumor cells overexpressing SSTR2. In our study, we investigated the role of receptor expression levels in receptor-mediated endocytosis, focusing on PDAC cell lines PANC-1, MiaPaca-2, and CFPAC-1. Among the PDAC cell lines, CFPAC-1 exhibited the highest SSTR2 expression, while MiaPaca-2 showed the lowest. As predicted, our innovative delivery system, octreotide-DSPE-PEG-liposomal paclitaxel, has demonstrated impressive efficiency in administering loaded drugs to targeted tumor cells. The cytotoxicity studies revealed a significant finding; namely, the targeted delivery system most effectively inhibited tumor growth in CFPAC-1 cells, aligning with the cell line’s high SSTR2 expression levels. This finding indicates that the targeted system can deliver the drug more effectively to cells with higher receptor expression, leveraging receptor-mediated endocytosis for enhanced drug uptake. The effectiveness of our targeted drug delivery system was further validated through tumor inhibition studies conducted in animal models. The OCT-Lip-PTX-treated tumors showed remarkable control over tumor growth across both cell line groups. Notably, in the group derived from CFPAC-1 cells, which exhibited the highest SSTR2 expression levels, we observed a halt in tumor growth and actual tumor shrinkage. The results underscore the potential of our targeted delivery system to improve drug accumulation within SSTR2-positive tumor cells, thereby enhancing the therapeutic efficacy against cancer while reducing the likelihood of the adverse effects typically associated with nonspecific drug distribution, and not only inhibiting tumor progression but also inducing tumor regression in cases where receptor expression is maximally aligned with the targeting mechanism of the therapy. This targeted approach promises to direct more of the active drug to the tumor site, minimizing unintended interactions with healthy tissue and organs and offering a more efficient and safer cancer treatment strategy.

It should be stressed that different composition of targeted and non-targeted formulations theoretically could affect their pharmacokinetics. However, the primary influence on the pharmacokinetics and distribution of the delivery system is provided by active targeting specifically to cancer cells. Previously, we revealed that targeting nanocarriers to tumor-specific receptors minimizes the influence of the architecture, composition, size, and molecular mass of nanocarriers on the efficacy of cancer treatment [30]. By comparing three types of carriers containing PTX (linear PEG polymer, polyamidoamine (PAMAM) dendrimers, and PEGylated liposomes similar to the present study), we showed that the pharmacokinetics and distribution of all cancer-targeted delivery systems were similar despite the different chemical compositions and architecture of nanoparticles. Consequently, the relatively small differences in the composition of non-targeted and targeted liposomes should have minimal influence on these parameters compared to the targeting itself.

The crucial question that demands further exploration is how to reconcile the limited effect observed in vitro with the significant differences observed in vivo regarding tumor growth delay. This is a typical situation when non-targeted and cancer-targeted systems are investigated both in vitro and in vivo [44]. The improvement of cellular internalization (observed in vitro) is only one, and not the primary, advantage of active tumor targeting by using ligands for the receptors expressed in cancer cells. The main mechanism of enhancing the anticancer activity of tumor-targeted formulations is a dramatic change in the organ distribution of targeted formulations. In addition to the tumor, non-targeted nanotechnology-based formulations accumulate in the liver, kidney, and spleen [30,44]. Their accumulation in tumors is attributed to the so-called enhanced permeability and retention (EPR) effect (passive targeting), leading to the retention of high-molecular-weight substances in extremely vascularized tumors with limited and impaired lymphatic drainage. In contrast, tumor-targeted formulations predominately accumulate in the tumor, leaving healthy organs intact. Therefore, in most cases, tumor-targeted systems demonstrate much more pronounced augmentation of their antitumor efficacy compared to their non-targeted counterpart in vivo than in vitro.

## 4. Materials and Methods

### 4.1. Chemical and Reagents

Paclitaxel (TAX) and Tween-80 were purchased from Sigma (St. Louis, MO, USA). Egg phosphatidylcholine (Egg PC), Cholesterol, and DSPE-PEG2000-azide (1,2-distearoyl-sn-glycero-3-phosphoethanolamine-N-[azido (polyethylene glycol)-2000]) were purchased from Avanti Polar Lipids (Alabaster, AL, USA). To create a somatostatin analog, octreotide (H-D-Phe-Cys*-Phe-D-Trp-Lys-Thr-Cys*-Thr-ol) 5-pentynecarboxylic acid was used to replace the N-terminus of the D-Phenylalanine group with an alkyne group. The resulting 5-pentynecarbonyl-octreotide was synthesized by Peptides International, Inc. (Louisville, KY, USA). Fetal bovine serum (FBS), 3-(4,5-dimethylthiazol-2-yl)-2,5-diphenyl tetrazolium bromide (MTT) reagents and dimethyl sulfoxide (DMSO) were purchased from Sigma (St. Louis, MO, USA). Horse serum was obtained from Thermo Fisher Scientific (Waltham, MA, USA). SSTR2 primers were purchased from Invitrogen (Waltham, MA, USA). Anti-SSTR2 primary antibodies were obtained from Santa Cruz Biotechnology (Dallas, TX, USA). Horseradish peroxidase (HRP)-conjugated secondary antibody was purchased from Cell Signaling Technology (Danvers, MA, USA).

### 4.2. Cell Lines and Culture Conditions

Human pancreatic cancer cell lines PANC-1, MiaPaca-2, and CFPAC-1 were chosen based on the literature reports about their expression of SSTR2, and all cell lines were purchased from the American Type Culture Collection (ATCC, Manassas, VA, USA). PANC-1 cells were cultured in T25 flasks using Dulbecco’s modified Eagle medium (DMEM), supplemented with 10% FBS and 1% penicillin-streptomycin solution (10,000 I.U./mL penicillin, 10,000 (μg/mL) streptomycin). MiaPaca-2 cells were also cultured in T25 flasks using Dulbecco’s Modified Eagle Medium (DMEM), supplemented with 10% FBS, 2.5% horse serum, and 1% penicillin–streptomycin solution. CFPAC-1 cells were grown in Iscove’s modified Dulbecco’s medium (IMDM), supplemented with 10% FBS and 1% penicillin–streptomycin solution. All the cells were cultured at 37 °C with humidity control in a CO_2_ incubator.

### 4.3. Expression of SSTR2 mRNA

To measure the gene expression of SSTR2, all three cell lines were incubated in cell medium in T-75 flasks and harvested until they reached 70% confluence. Then, total RNA was extracted from three cell lines using RNeasy^®^ Mini Kits (Qiagen, Valencia, CA, USA) according to the manufacturer’s instructions. The extracted mRNA was reverse-transcribed into cDNA using High-Capacity cDNA Reverse Transcription Kits (Applied Biosystems, Carlsbad, CA, USA) with a Veriti™ 96-well thermal cycler (Applied Biosystems, Carlsbad, CA, USA). Finally, the cDNA levels for SSTR2 in PANC-1, MiaPaca-2, and CFPAC-1 cells were measured using QRT-PCR with the Step One Plus System (Applied Biosystems, Carlsbad, CA, USA).

### 4.4. Expression of SSTR2 Protein

Immunocytochemistry (ICC) images were used to visualize the SSTR2 protein expression. Cells were seeded in 0.01% poly-L-lysine treated 4-well chamber slides. After 24 h of incubation, the media was removed, and the cells were washed with phosphate-buffered saline (PBS). Then, the cells were fixed with 4% paraformaldehyde and permeabilized with methanol. Next, after washing with phosphate-buffered saline with Tween 20 (PBS-T) buffer, the cells were blocked with 1% bovine serum albumin prepared in PBS and incubated in anti-SSTR2 primary antibodies. Then, the cells were incubated with Alexa Fluor 488-conjugated secondary antibodies. Finally, the cells were stained with 4,6-diamidino-2-phenylindole (DAPI) (Sigma Chemical Co., St. Louis, MO, USA) and mounted with ProLong Gold Antifade Mountant (Thermo Fisher Scientific, Waltham, MA, USA). Images were recorded using a 10× objective of a fluorescence microscope.

The SSTR2 protein expression levels were measured by Western blotting. Briefly, cells were seeded into 6-well plates. Then, all culture media were cleared, and each well was washed twice with PBS. Next, cells were treated with ice-cold radioimmunoprecipitation assay (RIPA) buffer, supplemented with Triton X-100, a protease and phosphatase inhibitor cocktail, for 25 min and sonicated in ice water for 1 min before being transferred into microcentrifuge tubes. Cell lysates were then centrifuged at 10,000× *g* for 10 min at 4 °C, and the supernatant was transferred into new microcentrifuge tubes. The protein was quantified with a Pierce bicinchoninic acid (BCA) protein assay (Pierce, Rockford, IL, USA). Then, 40 μg of proteins were added into each well of 4 to 12% NuPage Bis-Tris gel (Invitrogen, Waltham, MA, USA) for electrophoresis for 50 min at 200 V. Proteins were transferred from the gel to PVDF membranes with an iBlot 2 dry blotting system (Thermo Fisher Scientific, Waltham, MA, USA). Next, the membranes were submerged in 5% non-fat milk with PBS-T (0.05% Tween 20 in PBS buffer) on a rotating shaker for 1 h at room temperature to block non-specific binding, followed by washing with PBS-T. Then, the membranes were incubated overnight at 4 °C in anti-SSTR2 primary antibodies (1:100 dilution, Santa Cruz Biotechnology, Dallas, TX, USA). GAPDH was used as a loading control. After that, membranes were washed three times with PBS-T and incubated in horseradish peroxidase (HRP)-conjugated secondary antibody for 2 h at room temperature. Finally, to develop protein bands, membranes were soaked in SuperSignal™ West Pico PLUS Chemiluminescent Substrate (Thermo Scientific, Waltham, MA, USA) for 10 min and visualized using the BIO-RAD ChemiDoc Imaging System (Bio-Rad Laboratories, Hercules, CA, USA).

A quantitative analysis of Western blotting and ICC images was performed using ImageJ software Version 1.53m (https://imagej.net/ij/, accessed on 19 May 2024).

### 4.5. Preparation and Characterization of OCT-DSPE-PEG_2000_

A copper-catalyzed azide–alkyne click chemistry (CuAAC) reaction [45] was performed to conjugate DSPE-PEG-Azide with 5-pentynecarbonyl-octreotide (Figure 2). In total, 50.0 mg of DSPE-PEG-Azide (MW 2816.48, 0.017 mmol) was dissolved in 3 mL tetrahydrofuran (THF) in a 10 mL RB flask. Next, 18.0 mg of 5-pentynecarbonyl-octreotide (MW 1098.47, 0.016 mmol) was dissolved in 2 mL of 1:1 THF and methanol and added to the RB flask while stirring. After that, 100 µL of 20 mM CuSO_4_ was added to 100 µL of 50 mM tris (benzyltriazolylmethyl) amine (THPTA), and 200 µL of this mixture was added dropwise to the reaction mixture and stirred for one minute. Next, 25 µL of 100 mM aminoguanidine was added to the reaction mixture and stirred for one minute for homogeneous mixing. Finally, 25 µL of 100 mM sodium ascorbate was added to this reaction mixture, and the reaction mixture was stirred at 500 rpm for 24 h at room temperature. After that, the reaction mixture was evaporated to dryness at room temperature to get the crude mixture of the product.

The crude reaction mixture obtained was dissolved in 1 mL of water and transferred to a 3.5 kDa molecular weight cut-off (MWCO) dialysis tube (Repligen, Waltham, MA, USA), which was run against 1.5 L of 0.04 (M) EDTA buffer (pH 8.2). The dialysis was run for 36 h, and the buffer was changed after 3, 6, 12, and 24 h. Then, the purified product was transferred into a 5 mL beaker and left at room temperature for 1 h to evaporate the remaining solvent. The final product was lyophilized to get the ‘DSPE-PEG_2000_-OCT Ligand’ in solid powder form.

The MALDI-TOF-MS analysis of the DSPE-PEG_2000_-OCT purified ligand was redissolved in MeOH to reach a 10 mg/mL concentration. The measurement was carried out using a matrix of 10 mg/mL sinapinic acid (SA) in 2.5 mg/mL of cyano-4-hydroxycinnamic acid (CHCA), 50% acetonitrile, and 0.1% trifluoroacetic acid (TFA), and 5 pM of insulin was used as the external calibration standard.

### 4.6. Preparation of PEGylated Liposomal Paclitaxel, OCT-PEGylated Liposomal Paclitaxel, and Fluorescent Liposomal Paclitaxel

Paclitaxel-loaded PEGylated liposomes were prepared using the previously developed procedure [31]. Briefly, in untargeted formulation, the liposomes were prepared with lipids in a molar ratio of 51:44:5 mole% of egg yolk phosphatidylcholine (EPC)/Cholesterol/1,2,-distearoyl-sn-glycero-3-phosphoethanolamine-N-aminopolyethelenglycol- 2000 ammonium salt (DSPE-PEG_2000_) and 0.878 mM paclitaxel. For the targeted formulation OCT-PEGylated Liposome Paclitaxel, the lipids were mixed in a molar ratio of 47:41:2:10 of EPC/Cholesterol/DSPE-PEG_2000_/DSPE-PEG-OCT with 0.878 mM of paclitaxel and dissolved in the same solvent. The fluorescent liposomal paclitaxel was formulated with 100 μg of Oregon Green R 488 Paclitaxel (green fluorescence) and 1 mg of Egg Liss Rhod PE (red MAL fluorescence), along with other lipids using the aforementioned method.

The lipids and paclitaxel mixture were dissolved in 4.0 mL of 3:1 chloroform/methanol. Once fully dissolved, the clear liquid was transferred into a 250 mL round-bottom flask and evaporated under reduced pressure at 37 °C on a rotary evaporator (Rotavapor^®^ R-210/R-215, BUCHI Corp., New Castle, DE, USA). The resulting thin layer was rehydrated with 4 mL of a 0.9% saline solution. To create unilamellar liposomes, the mixture was sonicated continuously for 15 min. The resulting liposome product was then transferred into 8 kD MWCO dialysis bags and dialyzed in 0.9% saline while stirring (100 rpm) for 12 h at 4 °C to remove any unentrapped, free paclitaxel. All purified products were stored at 4 °C for further studies.

### 4.7. Zeta Potential, Particle Size, and Polydispersity Index (PDI)

The particle size, zeta potential, and polydispersity index (PDI) of liposome samples were measured by Malvern Zetasizer Nano (Malvern Instruments, Malvern, UK) at 25 °C. An aliquot of 25 μL of each formulation sample was diluted with 1.5 mL of 0.9% saline in Malvern disposable cuvettes. All measurements were completed in triplicate, and average values were calculated.

### 4.8. Atomic Force Microscopy (AFM)

The shape of all types of liposomal formulations were studied by atomic force microscopy imaging using the previously described procedure [46]. Briefly, 50 μL of a liposome suspension in water was deposited on pre-cut (∼25 × 25 mm^2^) and pre-cleaned Plain Premium microscope slides (Fisher Scientific Co., Pittsburgh, PA, USA), kept for 10 min at 100% humidity to achieve particles precipitation. Water was removed by dry nitrogen flow and dried samples were subjected to imaging with an atomic force microscope (Nano-R AFM Pacific Nanotechnology Instrument, PNI, Santa Clara, CA, USA) in close contact (tapping) mode using tapping-mode-etched OMCLAC160TS silicon probes (Olympus Optical Co., Tokyo, Japan). The captioning was performed in height mode.

### 4.9. High-Performance Liquid Chromatography (HPLC) Analysis of Paclitaxel

A Waters HPLC system was utilized to determine the concentration of paclitaxel in the samples. The system was equipped with dual pumps (Waters 1525), a Waters 2487 Dual Absorbance UV detector, and a Waters 717 autosampler. Each sample was injected with 20 µL and run at a 0.5 mL/min flow rate. The samples were examined using a reverse-phase LiChrospher^®^100 RP-18 column (250 × 4 mm, 5 μm, Merck, Darmstadt, Germany) at room temperature. The detecting wavelength employed was 227 nm. The mobile phase comprised acetonitrile and water (60:40, *v*/*v*).

### 4.10. Entrapment Efficiency and HPLC Measurement

Dialysis was employed as a method to separate free paclitaxel from entrapped drugs. In total, 1 mL crude liposomal suspension, either Lip-PTX or OCT-Lip-PTX, was introduced into dialysis bags with a MWCO of 15 kDa. These bags were then dialyzed in 0.9% saline solution at a temperature of 4 °C for 12 h, with continuous stirring at 100 rpm to ensure consistent diffusion. An aliquot of 25 µL of the crude, or recovered liposome product after dialysis, was dissolved and diluted in 975 µL of the dissolving solvent composed of water, isopropanol, ether, and ethanol (5:2:1:2, *v*/*v*/*v*/*v*). The concentration of the paclitaxel added initially and the concentration of the paclitaxel entrapped were measured by HPLC, and the actual weight of the total paclitaxel before (*W_total_*) and after (*W_entrapped_*) purification can be calculated by multiplying proper dilution factors [47]. Drug entrapment efficiency was calculated by the equation below:

$$EE\%=\frac{W_{entrapped}}{W_{total}}\times 100\%$$

### 4.11. Paclitaxel Release Profile

An aliquot of 0.8 mL of liposome paclitaxel samples Lip-PTX or OCT-Lip-PTX was added to 15 kDa MWCO dialysis bags individually. The dialysis bags were then submerged in beakers containing 80 mL of 1% Tween 20/PBS solution, with magnetic stirring set to 100 rpm. The study was carried out at three different temperatures, 4 °C, 25 °C, and 37 °C, over 7 days. In total, 1 mL of samples were collected at specific time points of 0 h, 0.5 h, 1 h, 2 h, 3 h, 6 h, 8 h, 12 h, 24 h, 48 h, 72 h, 96 h, 120 h, 144 h, and 168 h. After sample collection, an equal volume of 1% Tween 20/PBS solution was replaced at each time point. As explained in the previous section, the samples were analyzed using HPLC methods.

### 4.12. Cellular Internalization Study

In order to visualize liposomes, they were labeled with near-infrared cyanine dye Cy5.5 (Amersham Biosciences, Piscataway, NJ, USA) with red fluorescence, as previously described [48]. Paclitaxel, Oregon Green™ 488 Conjugate (Fluotax) with green fluorescence (Thermo Fisher Scientific, Waltham, MA, USA) was used instead of regular PTX in the part of synthesized formulations to visualize drug release from the system inside cancer cells. The fluorescence of Fluotax tightly packed in liposomes was quenched. Therefore, the appearance of the green fluorescence signal inside the cells testified the release of the drug from liposomes inside the cells.

All three cell lines were individually plated in 4 well-chambered Coverglass (Thermo Scientific Nunc, Naperville, IL, USA) and allowed to incubate for 24 h at 37 °C. The culture medium for each formulation was the same (DMEM and IMDM for PANC-1 and CFPAC-1 cells, respectively). Following this, they were treated with fluorescent liposomal paclitaxel, which had been previously prepared with the same volume and concentration of PTX for non-targeted and targeted systems. After 24 h of treatment, the cells were rinsed with Dulbecco’s phosphate-buffered saline (DPBS) containing 0.05% Tween 20 and fixed with a 4% formaldehyde solution. Next, 0.5 mL of DPBS was added to each chamber. The cell nuclei were stained by a blue-fluorescent DNA stain 4′,6-diamidino-2-phenylindole (DAPI). The slides were observed using a Confocal Microscope (Leica TCS SP8, CarlZeiss, Jena, Germany), and pictures were captured and analyzed using LAS AF Lite Leica Version 2.6.3 software.

### 4.13. Cytotoxicity Study

MTT (3-(4,5-dimethylthiazol-2-yl)-2,5-diphenyl tetrazolium bromide) assays were used to measure the cell viability of PANC-1, MiaPaca-2, and CFPAC-1 cells after treatment. Cells were seeded in 96-well plates with 0.1 mL culture media in each well and incubated at 37 °C for 24 h. Then, culture media were removed, and the cells were, respectively, treated with paclitaxel (free paclitaxel), PEGylated liposomal paclitaxel, OCT-DSPE-PEGylated liposomal paclitaxel, or empty liposomes in 0.2 mL of various concentrations. Each treatment mentioned above was prepared in 100 µM, and 12 working solutions in decreasing concentrations were prepared by serial dilutions (1:10). Cells incubated in fresh culture media were used as a control. After 72 h of incubation, treatment solutions were removed, and cells were incubated in 0.12 mL of a 1 mg/mL MTT solution at 37 °C for 3 h. The Formazan crystals were dissolved with a precise reagent (10.5 g SDS in 25 mL dimethylformamide (DMF) + 25 mL deionized (DI) water). The absorbance was measured using Tecan Infinite M200 PRO (Morrisville, NC, USA) at a wavelength of 570 nm. The IC_50_, a commonly used parameter in assessing cytotoxicity, denotes the concentration of a given treatment that leads to a 50% inhibition of cell proliferation. Based on the results, CFPAC-1 and MiaPaca-2 cells were selected for the following in vivo studies and analysis.

### 4.14. In Vivo Animal Studies

Animal studies were performed according to the protocols and animal use procedures approved by the Institutional (Rutgers, the State University of New Jersey) Animal Care and Use Committee (IACUC). Male and Female NCr (nu/nu strain), 6–8-week-old mice, weighing about 20 g, were purchased from Taconic Farms, Inc. (Germantown, NY, USA) and were housed in cages under controlled laboratory conditions (temperature 22–24 °C, relative humidity 50 ± 10% and 12 h/12 h light/dark cycle) and allowed free access to a sterilized rodent pellet diet and acidified drinking water. The animals were acclimatized for at least 72 h before any experiments, and “pre-numbered” ear tags were used to identify each mouse.

#### 4.14.1. Maximum Tolerated Doses (MTDs) of Paclitaxel

For the Paclitaxel MTD study, healthy mice received one injection of the clinically used Taxol ^®^ formulation at 5 different concentrations. For each concentration tested, 5 mice were used. The animals were observed for signs of acute toxicity, such as weight loss and abnormal behavior.

For MTD studies, the concentrations (doses) of PTX in CrEL-D tested were 1, 2, 2.5, 3.0, and 3.5 mg PTX/kg in 0.2 mL injection per animal *n* = 5 per dose). Healthy mice were weighed and then began receiving IV tail injections (~0.2 mL per mouse) of different concentrations of CrEL-D using a q5dx4 schedule. Mice were monitored and weighed on the day of the treatment, the day after, and every other day during the trial. Toxicity was assessed as a percentage of weight loss. The MTD was defined as the highest dose with <15% body weight loss and not causing significant lethality or any prominent observable changes during the trial. The difference in mean body weight was calculated concerning the beginning of treatment (day 1) as follows:

(mean body weight on day x − mean body weight on day 1)/(mean body weight on day 1) × 100

The dose of paclitaxel was determined to be the maximum tolerated dose and was used in all studied drug formulations.

#### 4.14.2. Animal Model of Pancreatic Cancer and Antitumor Activity

An animal model of human pancreatic cancer xenografts was created by injecting 5 × 10^6^ CFPAC-1 and Mia PaCa-2 cells subcutaneously into the athymic nu/nu mice flanks. When the tumors reached a size of about 0.3–0.4 cm^3^, mice were randomly divided into groups (6 animals per group) and treated intraperitoneally with five different formulations: saline (control), empty liposomes, Raxol^®^, PEGylated liposomal paclitaxel, OCT-DSPE-PEGylated liposomal paclitaxel. The animals were treated twice per week for 4 weeks. The tumor growth was measured by a caliper on the day of the treatment, the day after, and every other day during the trial. Tumor volume was calculated as d^2^ × D/2, where d and D are the smallest and widest diameter of the tumor in mm, respectively. According to the approved institutional animal use protocol, the mice were sacrificed when the tumor reached around 1.2–1.3 cm^3^. All other measurements were performed 24 h after the treatment. Changes in tumor size were used as an overall marker for antitumor activity.

## 5. Conclusions

A chemically modified somatostatin analog-conjugated to paclitaxel-loaded liposomes was formulated for the chemotherapy of pancreatic cancer. Based on our studies, this novel formulation displayed high selectivity to SSTR2-expressing tumor cells, significantly inhibited cell proliferation, and increased drug cytotoxicity, demonstrating superior tumor suppression effects compared to the non-targeting formulations, with the possibility of maintaining limited toxicity on healthy organs. Therefore, the novel 5-pentynecarbonyl-octreotide-conjugated liposomal paclitaxel presents a promising delivery system specific to pancreatic cancer cells and tissues with high expression of SSTR2.

While paclitaxel serves as a model drug in this study, various cytotoxic agents such as doxorubicin, methotrexate, camptothecin, carboplatin, cisplatin can also be incorporated into the proposed delivery system for co-encapsulation, given the amphiphilic structure of the liposomal membrane (for lipophilic drugs) and their liquid inner space (for hydrophilic drugs). The use of hydrophilic drugs can potentially improve the drug-loading capacity and stability of liposomes. However, even for the hydrophobic PTX, we registered close to 90% loading capacity and decent stability of the formulations under short-term storage in the refrigerator, overall. In general, the stability of lyophilized lipid-based formulations is excellent, and changes in nanoparticle characteristics, including the drug-loading capacity, are minimal after one freezing/thaw cycle. In contrast, at body temperature, after entering cancer cells, liposomes should release the encapsulated drug in order to induce the death of cancer cells. Consequently, the further enhancement of the stability of carriers under body temperature would decrease their ability to kill cancer cells.

An additional optimization can benefit liposomal formulations concerning their composition, PEGylated lipids and OCT fractions, and paclitaxel loading, among other factors. The main objective of the present research was to demonstrate that targeting a nanotechnology-based drug formulation to pancreatic cancer cells using a somatostatin analog could increase its effectiveness against tumors while drawing attention to this approach. In our laboratory, we are exploring further optimization of the tumor-targeted delivery of anticancer drugs to pancreatic cancer.

The present experimental data show that the cancer-targeted liposomal formulations of anticancer drugs are more toxic when compared with non-targeted formulations and free-non-bound drugs in the same concentration. Our previous detailed investigations of various delivery systems clearly showed that tumor targeting almost eliminates adverse toxic side effects on healthy organs and tissues in vivo [9,30,44]. Based on these results, we do not expect that the proposed approach in the current investigation to pancreatic tumor-targeting can increase the adverse side effects compared to similar liposomal formulations targeted to tumors by the LHRH peptide. However, further toxicologic evaluation of the proposed system is required and is being conducted in our lab.

Due to the complex and heterogeneous nature of PDAC’s tumor microenvironment, employing multifunctional nanoparticles that combine tumor-penetrating peptides such as TAT, antennapedia, and iRGD could possibly improve the precision and efficiency of targeting, leading to enhanced intracellular delivery of therapeutic agents. Combining the targeting nano-based carriers with gene-silencing approaches, such as RNAi tailored to the specific genetic makeup of patients, holds considerable promise for advancing personalized medicine [6]. This approach could markedly improve therapeutic outcomes and benefit patients with various genetic profiles. Nonetheless, translating these promising preclinical findings into clinical practice requires further investigation, including the optimization of conjugate design, comprehensive in vivo efficacy and toxicity evaluations, and, ultimately, clinical trials to ascertain their safety and effectiveness in humans.

## Figures and Tables

**Table 1:** Characterization of empty liposomes (Lip), targeted liposomes without the drug (OCT-Lip), non-targeted liposomes containing paclitaxel (OCT-Lip-PTX), and tumor-targeted liposomes containing paclitaxel (OCT-Lip-PTX). Means ± SD are shown.

Formulation	Particle Size, nm	Polydispersity Index	Zeta Potential, mV	Encapsulation Efficiency, %
Lip	88.76 ± 0.09	0.210 ± 0.008	−2.22 ± 1.36	88.76
OCT-Lip	96.10 ± 0.73	0.261 ± 0.008	−1.78 ± 0.07	96.10
Lip-PTX	134.20 ± 0.93	0.248 ± 0.007	−3.20 ± 0.72	94.4
OCT-Lip-PTX	111.30 ± 0.31	0.230 ± 0.002	−3.27 ± 0.56	88.46

## Data Availability

The data supporting the conclusions of this article will be made available by the authors on request.
